# Gut microbiome is not associated with mild cognitive impairment in Parkinson’s disease

**DOI:** 10.1038/s41531-024-00687-1

**Published:** 2024-04-06

**Authors:** Velma T. E. Aho, Matthias Klee, Zied Landoulsi, Anna Heintz-Buschart, Lukas Pavelka, Anja K. Leist, Rejko Krüger, Patrick May, Paul Wilmes, on behalf of the NCER-PD Consortium, Geeta Acharya, Gloria Aguayo, Myriam Alexandre, Muhammad Ali, Wim Ammerlann, Giuseppe Arena, Michele Bassis, Roxane Batutu, Katy Beaumont, Sibylle Béchet, Guy Berchem, Alexandre Bisdorff, Ibrahim Boussaad, David Bouvier, Lorieza Castillo, Gessica Contesotto, Nancy De Bremaeker, Brian Dewitt, Nico Diederich, Rene Dondelinger, Nancy E. Ramia, Angelo Ferrari, Katrin Frauenknecht, Joëlle Fritz, Carlos Gamio, Manon Gantenbein, Piotr Gawron, Laura Georges, Soumyabrata Ghosh, Marijus Giraitis, Enrico Glaab, Martine Goergen, Elisa Gómez De Lope, Jérôme Graas, Mariella Graziano, Valentin Groues, Anne Grünewald, Gaël Hammot, Anne-Marie Hanff, Linda Hansen, Michael Heneka, Estelle Henry, Margaux Henry, Sylvia Herbrink, Sascha Herzinger, Alexander Hundt, Nadine Jacoby, Sonja Jónsdóttir, Jochen Klucken, Olga Kofanova, Rejko Krüger, Pauline Lambert, Roseline Lentz, Laura Longhino, Ana Festas Lopes, Victoria Lorentz, Tainá M. Marques, Guilherme Marques, Patricia Martins Conde, Deborah Mcintyre, Chouaib Mediouni, Francoise Meisch, Alexia Mendibide, Myriam Menster, Maura Minelli, Michel Mittelbronn, Saïda Mtimet, Maeva Munsch, Romain Nati, Ulf Nehrbass, Sarah Nickels, Beatrice Nicolai, Jean-Paul Nicolay, Fozia Noor, Clarissa P. C. Gomes, Sinthuja Pachchek, Claire Pauly, Laure Pauly, Lukas Pavelka, Magali Perquin, Achilleas Pexaras, Armin Rauschenberger, Rajesh Rawal, Dheeraj Reddy Bobbili, Lucie Remark, Ilsé Richard, Olivia Roland, Kirsten Roomp, Eduardo Rosales, Stefano Sapienza, Venkata Satagopam, Sabine Schmitz, Reinhard Schneider, Jens Schwamborn, Raquel Severino, Amir Sharify, Ruxandra Soare, Ekaterina Soboleva, Kate Sokolowska, Maud Theresine, Hermann Thien, Elodie Thiry, Rebecca Ting Jiin Loo, Johanna Trouet, Olena Tsurkalenko, Michel Vaillant, Carlos Vega, Liliana Vilas Boas, Paul Wilmes, Evi Wollscheid-Lengeling, Gelani Zelimkhanov

**Affiliations:** 1 Luxembourg Centre for Systems Biomedicine, University of Luxembourg, Esch-sur-Alzette, Luxembourg; 2 Institute for Research on Socio-Economic Inequality (IRSEI), Department of Social Sciences, University of Luxembourg, Esch-sur-Alzette, Luxembourg; 3 Swammerdam Institute of Life Sciences at University of Amsterdam, Amsterdam, the Netherlands; 4 Parkinson’s Research Clinic, Centre Hospitalier de Luxembourg, Luxembourg, Luxembourg; 5 Transversal Translational Medicine, Luxembourg Institute of Health, Strassen, Luxembourg; 6 Department of Neurology, Centre Hospitalier de Luxembourg, Luxembourg, Luxembourg; 7 Department of Life Sciences and Medicine, Faculty of Science, Technology and Medicine, University of Luxembourg, Esch-sur-Alzette, Luxembourg; 8 Luxembourg Institute of Health, Strassen, Luxembourg; 9 Centre Hospitalier de Luxembourg, Strassen, Luxembourg; 10 Centre Hospitalier Emile Mayrisch, Esch-sur-Alzette, Luxembourg; 11 Laboratoire National de Santé, Dudelange, Luxembourg; 12 Association of Physiotherapists in Parkinson’s Disease Europe, Esch-sur-Alzette, Luxembourg; 13 Faculty of Science, Technology and Medicine, University of Luxembourg, Esch-sur-Alzette, Luxembourg; 14 Department of Epidemiology, CAPHRI School for Public Health and Primary Care, Maastricht University Medical Centre+, Maastricht, the Netherlands; 15 Private practice, Ettelbruck, Luxembourg; 16 Parkinson Luxembourg Association, Leudelange, Luxembourg; 17 Luxembourg Center of Neuropathology, Dudelange, Luxembourg; 18 Department of Life Sciences and Medicine, University of Luxembourg, Esch-sur-Alzette, Luxembourg; 19 Private practice, Luxembourg, Luxembourg

**Keywords:** Microbiology, Parkinson's disease, Molecular biology

## Abstract

Gut microbiome differences between people with Parkinson’s disease (PD) and control subjects without Parkinsonism are widely reported, but potential alterations related to PD with mild cognitive impairment (MCI) have yet to be comprehensively explored. We compared gut microbial features of PD with MCI (*n* = 58) to cognitively unimpaired PD (*n* = 60) and control subjects (*n* = 90) with normal cognition. Our results did not support a specific microbiome signature related to MCI in PD.

Mild cognitive impairment (MCI) is a non-motor symptom of Parkinson’s disease (PD) that represents a risk factor for developing dementia, and can significantly impact quality of life.^1^ While gut microbial community differences between people with PD and individuals without parkinsonism are well established^2–7^, only a single publication has investigated the gut microbiome in PD with MCI, suggesting significant differences in several taxa when contrasting PD with MCI to PD with unimpaired cognition or to control subjects.^8^ To investigate whether these results could be replicated in a larger, geographically distinct cohort, we performed similar comparisons using data from the Luxembourg Parkinson’s Study^4^.

Our dataset comprised 58 people with PD and MCI (PD-MCI), 60 people with PD without cognitive impairment (PD-NC), and 90 cognitively normal control subjects. While there were differences in demographic and clinical variables between the control and PD groups, including that controls were younger and had lower frequency of constipation, the PD-MCI and PD-NC groups had similar profiles (Table 1).

**Table 1:** Demographic and clinical characteristics of study subjects

Characteristic^a^	Control, *N* = 90^b^	PD-NC, *N* = 60^b^	PD-MCI, *N* = 58^b^	*p*-value^c^	Control vs. PD-MCI^d^	Control vs. PD-NC^d^	PD-NC vs. PD-MCI^d^
Female sex	39 (43%)	20 (33%)	19 (33%)	0.315			
Constipation	6 (6.7%)	25 (42%)	28 (48%)	<0.001			
Age (years)	68.9 (66.1, 72.5)	71.3 (69.2, 74.9)	73.1 (68.8, 77.9)	0.002	0.001	0.078	0.162
MoCA	28 (27, 29)	28 (27, 29)	23 (22, 25)	<0.001	<0.001	0.777	<0.001
Body mass index	26.8 (24.1, 29.3)	27.6 (24.1, 30.3)	27.7 (25.3, 31.3)	0.055	0.060	0.276	0.427
Years of education	14 (11, 17)	14 (12, 17)	12 (10, 15)	0.061	0.092	0.714	0.092
Caffeine use	84 (93%)	53 (88%)	55 (95%)	0.449			
Spouse in current data	7 (7.8%)	4 (6.7%)	1 (1.7%)	0.309			
Probiotics in last 6 months	3 (3.3%)	2 (3.3%)	1 (1.7%)	>0.999			
Antibiotics in last 6 months	11 (12%)	9 (15%)	3 (5.2%)	0.211			
Vegan or vegetarian diet	2 (2.2%)	3 (5.0%)	2 (3.4%)	0.638			
Years since PD diagnosis		5 (3, 9)	4 (2, 8)	0.400			
Levodopa equivalent daily dosage (mg/day)		475 (339, 806)	472 (300, 874)	0.988			
MDS-UPDRS III		34 (21, 42)	32 (26, 43)	0.833			
Hoehn and Yahr stage		2.00 (2.00, 2.50)	2.00 (2.00, 2.50)	0.978			

^a^*MoCA* Montreal Cognitive Assessment score, *MDS-UPDRS* Movement Disorder Society Unified Parkinson’s Disease Rating Scale.

^b^Categorical variables: *n* (%); continuous variables: median (IQR).

^c^Categorical variables: Pearson’s Chi-squared test or Fisher’s exact test; Continuous variables: one-way ANOVA; PD-only continuous variables: Welch two sample t-test.

^d^pairwise t-test with Holm multiple comparison correction.

We did not observe any difference between the PD-MCI, PD-NC, and control groups in microbial community richness and evenness (alpha diversity) when tested without confounders (Fig. 1a, b, Supplementary Table 1a). In a linear regression model for the inverse Simpson index, including the three groups and potential confounding variables, both PD groups tended to have lower diversity than controls (0.1 > *p* > 0.05; Supplementary Table 1b). In a within-PD model with confounders, there was no difference between PD with or without MCI (Supplementary Table 1c).

**Fig. 1:**
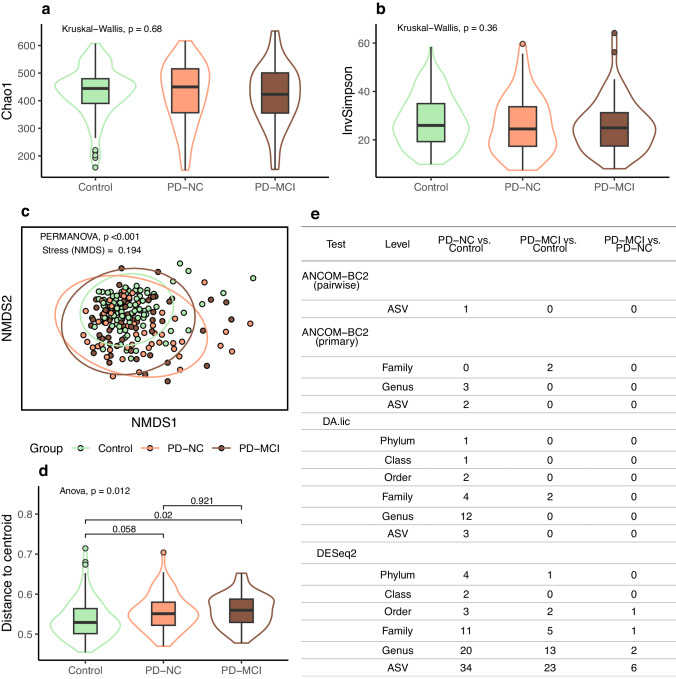
Microbial diversity and differential abundance comparisons for Parkinson’s disease with and without mild cognitive impairment. **a** Boxplot for richness (Chao1). **b** Boxplot for richness and evenness (inverse Simpson). **c** Community composition visualized as NMDS ordination of Bray-Curtis dissimilarity; ellipses indicate 95% confidence intervals. **d** Boxplot for groupwise distances to centroid from the ordination, with significances for pairwise comparisons from Tukey HSD test. **e** Numbers of differentially abundant taxa (multiple comparison corrected *p* < 0.05). In boxplots, box hinges represent the 1st and 3rd quartiles, whiskers range from hinge to the highest and lowest values that are within 1.5*IQR of the hinge, and outlines represent data distributions.

In comparisons of community composition (beta diversity), there was a difference between the three groups when tested with or without confounding variables (*p* < 0.001 for both) (Fig. 1c, Supplementary Tables 2a, b). Pairwise tests between controls and each of the PD groups also showed a significant group effect, but a within-PD test indicated no difference in relation to MCI status (Supplementary Tables 2c–e). In tests of sample dispersions between the groups, the difference was significant between PD-MCI and controls (*p* < 0.05), close to significant between PD-NC and controls (0.1 > *p* > 0.05) and not significant between PD-MCI and PD-NC (Fig. 1d; Supplementary Tables 2f, g).

We performed differential abundance comparisons with three tools: DESeq2^9^ and ANCOM-BC2^10^, commonly used methods with different statistical backgrounds, and DA.lic from the DAtest^11^ package, selected based on its performance compared to other tests (Supplementary Fig 1a–c, Supplementary Table 3a). Comparing controls to the PD groups resulted in many significant taxonomic clades when comparing either PD-MCI or PD-NC to controls (Fig. 1e, Supplementary Fig 1d, Supplementary Table 3b–d). Taxa which were significant with more than one test included, among others, decreased abundances of the family *Lachnospiraceae*, *Clostridiaceae* and *Butyricicoccaceae* in PD, and increases in *Enterobacteriaceae* and the genera *Hungatella* and DTU089 (family *Ruminococcaceae*). DESeq2 indicated increases in many additional taxa, such as the genera *Escherichia/Shigella* and *Methanobrevibacter*. However, when comparing PD-MCI to PD-NC, two out of three tests detected no significant taxa (Fig. 1e). DESeq2 highlighted 10 significant taxa for this comparison, notably less than in comparisons between other groups (Supplementary Fig 2a). Among the most significant taxa were genus *Streptococcus* (increased in PD-MCI) and an Amplicon Sequence Variant (ASV) classified as *Akkermansia muciniphila* (decreased in PD-MCI) (Supplementary Fig 2b–e).

Many of the taxa detected as differentially abundant between the PD and control groups were in line with previous publications, including the increased abundances of *Enterobacteriaceae*^7^*, Hungatella*^5,6^ and *Methanobrevibacter*^6^, and decreased abundances of *Lachnospiraceae*^5–7^ and *Butyricicoccaceae*^6,7^ in PD. The differences in beta diversity between control and PD subjects were also in line with the literature.^2–4,6,7^ As for comparisons related to PD with MCI, the previous publication on the topic reported a significant difference in beta diversity between PD-NC and PD-MCI, higher abundances of two families and four genera in PD-MCI compared to either PD-NC or controls, and decreases in two genera when contrasting PD-MCI and PD-NC.^8^ In our study, there was no difference in beta diversity between PD with and without MCI. When comparing specific taxa, only one of three tests indicated any differences between PD with and without MCI, and none of those taxa overlapped with the previous publication^8^. Considering the lack of overlap, the two studies do not suggest a consistent microbial signature representative of MCI in PD. The most compelling taxon detected in the present study was an *A. muciniphila* ASV, which was almost entirely absent in PD-MCI. *A. muciniphila* is typically increased in PD^3–7^, and more research regarding the significance of this taxon in PD and its subtypes is warranted.

To conclude, our comparisons reproduced previously detected differences between PD and control subjects but did not lend support to microbial community patterns specific to PD with MCI.

## Methods

Subject recruitment, faecal sample collection and processing as well as amplification and sequencing of the 16 S rRNA gene (regions V3–V4) have been described previously^4^. Participants were included in the present study if they matched UK Parkinson’s Disease Society Brain Bank clinical diagnostic criteria^12^ for typical PD; subjects with atypical or not yet specified parkinsonism were excluded. Control subjects genetically related to participants with PD were also excluded. The Luxembourg Parkinson’s Study^13^ was conducted according to the Declaration of Helsinki, with approval from the National Ethics Board (CNER Ref: 201407/13) and Data Protection Committee (CNPD Ref: 446/2017). All participants signed written informed consent.

MCI was defined according to Movement Disorder Society (MDS) taskforce criteria^14^, using a validated scale for cognitive assessment in PD (Montreal Cognitive Assessment^15^; MoCA), and information about the impact of cognitive impairment on daily living. For MoCA, the cutoffs used were <26 and >20. The impact of cognitive impairment was evaluated by MDS-UPDRS (Unified Parkinson’s Disease Rating Scale) question 1.1., assessed by a study physician, neuropsychologist, or PD specialized nurse during a semi-structured interview with the participant, together in discussion with family members, where possible. Constipation was defined based on Rome III criteria^16^. Levodopa equivalent daily dosage (LEDD) was calculated based on published conversion factors^17^.

Sequence data was processed with dadasnake^18^. Primers were removed using cutadapt^19^ allowing 20% mismatches and no indels. Quality filtering, ASV (Amplicon Sequence Variant) generation and chimera removal were performed using DADA2^20^. Reads were truncated at positions with Phred score < 10, or at 240 bp. Quality filtering was set to keep sequences with a maximum expected error of 2 and length of 240 bp. Downsampling was performed to 25,000 reads using seqtk^21^; samples with fewer reads were removed. ASVs were generated in pooled mode using default parameters. A minimum overlap of 12 bp was required for merging forward and reverse ASVs. Chimeric sequences were removed based on the consensus algorithm. Taxonomic classification was performed against SILVA v. 138^22^ using the naïve Bayesian classifier implemented in mothur^23^.

After excluding subjects that did not match diagnostic criteria, control subjects related to PD subjects, and subjects lacking microbiome data, the data contained results from 468 individuals. Additional exclusions were implemented as follows:

1. 236 subjects with age <= 64 years due to overrepresentation of younger individuals in the control group,
2. 3 subjects due to missing information on education or body mass index (BMI),
3. 5 subjects due to having <10,000 sequence reads left after removing rare ASVs (present in <10% of samples) and ASVs classified as chloroplasts or mitochondria,
4. 16 subjects due to reported use of corticosteroids or immunosuppressants in the past 6 months.

After these exclusions, the final data set used for analyses consisted of data from 208 individuals.

Statistical comparisons and visualizations were performed in R^24^ (version 4.3.2), using renv^25^ (1.0.3) for package management and knitr^26^ (1.43) for reporting. Comparisons of demographic and clinical variables were performed using Pearson’s Chi-squared test or Fisher’s exact test (categorical variables), Welch two sample t-tests (PD-only continuous variables), and one-way ANOVA with post-hoc two-tailed pairwise t-tests and “holm” multiple comparison correction (continuous variables).

Alpha diversity indices were calculated with phyloseq^27^ (1.42.0) and compared with Wilcoxon rank sum tests (variables with two categories), Kruskal-Wallis tests (variables with more than two categories) or Pearson correlations (continuous variables) as well as linear regression to model multiple variables together. Beta diversity was explored using phyloseq and vegan^28^ (2.6–4), with data subsampled to the lowest sequence read count in a sample. Bray-Curtis dissimilarity was used as the dissimilarity measure, visualizations were performed with Non-Metric Multidimensional Scaling (NMDS), and statistical comparisons with PERMANOVA (function: adonis2) using 9999 permutations. Confounder-corrected adonis2 models were run with the option ‘by = “margin”’ to calculate marginal effects. Additional tests for beta diversity included ANOVA and Tukey-HSD for multivariate homogeneity of group dispersions.

Differential abundance comparisons were performed with DESeq2^9^ (1.38.3), ANCOM-BC2 from ANCOMBC^10^ (2.1.4), and DA.lic, which implements LIMMA^29^ with CLR transformed data, from DAtest^11^ (2.8.0). DA.lic was selected based on the results of testDA from DAtest, which was run 50 times for each method, contrasting PD-NC to PD-MCI with a confounder-corrected model, using PD-only genus and ASV level data (ASVs trimmed to those present in > 30 samples). The final choice was based on test score > 0, false discovery rate < 0.25, and higher power than other similarly performing tests.

The model used for all differential abundance tests was taxon ~ Group (control/PD-NC/PD-MCI)+Sex+Age+BMI+Antibiotic use in past 6 months (yes / no)+Constipation (yes / no) + Education (categorical, split by median). DESeq2 was run with default parameters except for ‘sfType = “poscounts”’, and results were retrieved for all pairwise comparisons between the three subject groups. DA.lic and ANCOM-BC2 were run twice for each taxonomic level:

1. with full data and all three groups, for which these tests only provide results in relation to the reference level (only PD-NC vs. control and PD-MCI vs. control, not PD-MCI vs. PD-NC),
2. with PD-only data for the PD-MCI vs. PD-NC comparison.

DA.lic was performed using default settings. With ANCOM-BC2, the full data comparison (1) was additionally performed using the pairwise approach, which provides results for all three pairwise comparisons, but is more stringent. Additional parameters included setting ‘prv_cut’ to 0 and multiple comparison correction to “fdr” for both the default test and the pairwise test.

### Reporting summary

Further information on research design is available in the Nature Research Reporting Summary linked to this article.

### Supplementary information

Supplementary Information

Reporting Summary

## Data Availability

Patient data used in the preparation of this manuscript were obtained from the National Centre of Excellence in Research on Parkinson’s Disease (NCER-PD). NCER-PD datasets are not publicly available, as they are linked to the Luxembourg Parkinson’s Study and its internal regulations. The NCER-PD Consortium is willing to share its available data. Its access policy was devised based on the study ethics documents, including the informed consent form, as approved by the national ethics committee. Requests to access datasets should be directed to the Data and Sample Access Committee via email: request.ncer-pd@uni.lu.

## Code availability

The R code for this study is available at https://gitlab.lcsb.uni.lu/ESB/ncer-mci-microbiome.
