# Supplementary material for: Gut microbiome is not associated with mild cognitive impairment in Parkinson’s disease
Source: NPJ Parkinsons Dis. 2024 Apr 6;10:78. doi: 10.1038/s41531-024-00687-1 (PMC10998870; doi:10.1038/s41531-024-00687-1)
Supplement: Supplementary file 1 — Supplementary Information [file 41531_2024_687_MOESM1_ESM.pdf]

Supplementary Information for

Gut microbiome is not associated with mild cognitive impairment in Parkinson's disease

Supplementary Figure 1

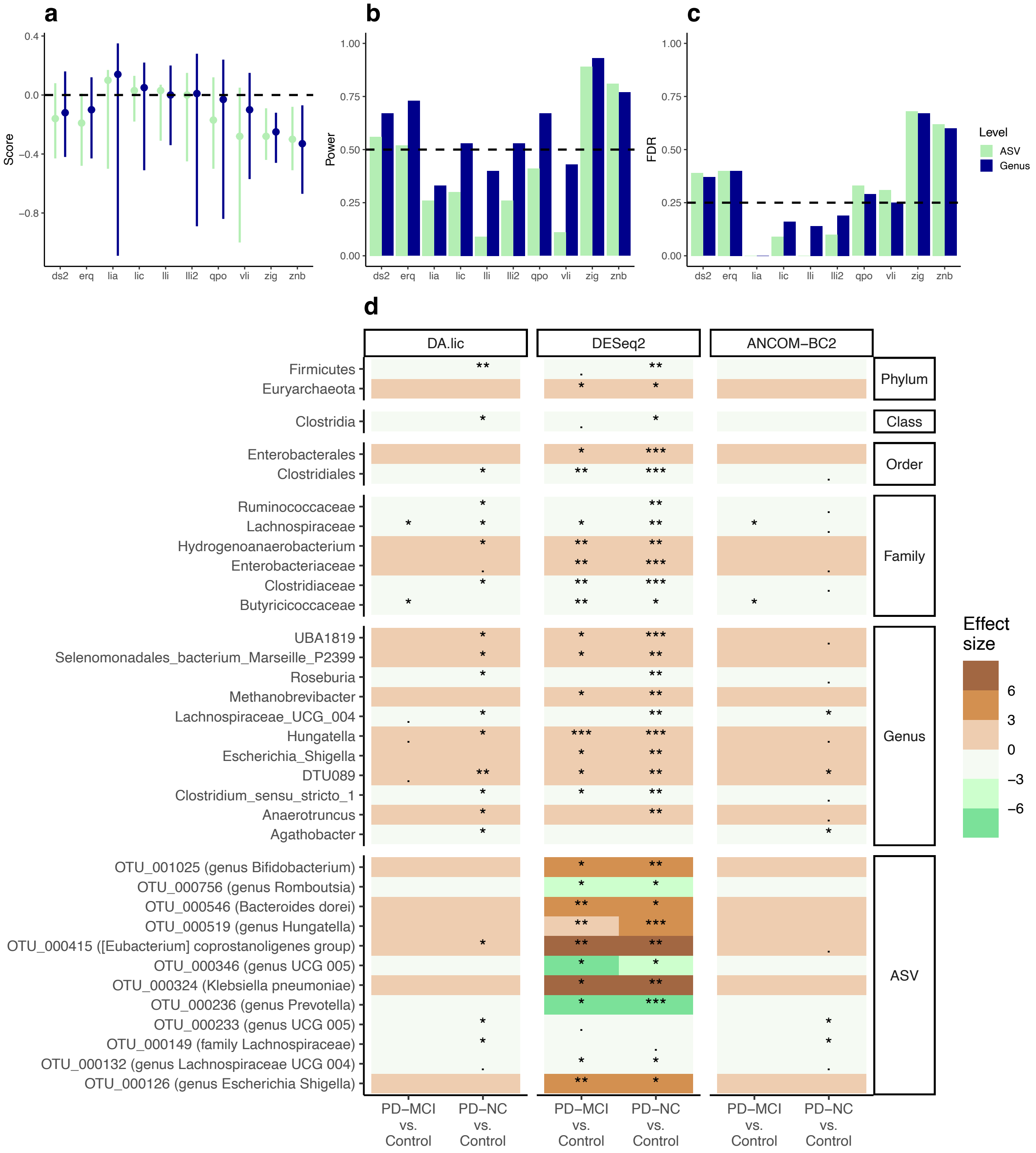

a-c. Results of differential abundance test comparisons with DAtest; **a.** Test score, points indicate mean and lines 90% confidence interval. **b.** Statistical power. **c.** False Discovery Rate. For test abbreviations, see Supplementary Table 3a. **d.** Heatmap summarizing taxa that were differentially abundant ($q < 0.05$) in at least 2 out of 6 possible result lists (2 contrasts [PD-MCI vs control, PD-NC vs control] and 3 tests [ANCOM-BC2, DESeq2, DA.lic from DAtest]). Symbols: · : $0.1 > q > 0.05$; * : $q < 0.05$; ** : $q < 0.01$; *** : $q < 0.001$.

Supplementary Figure 2

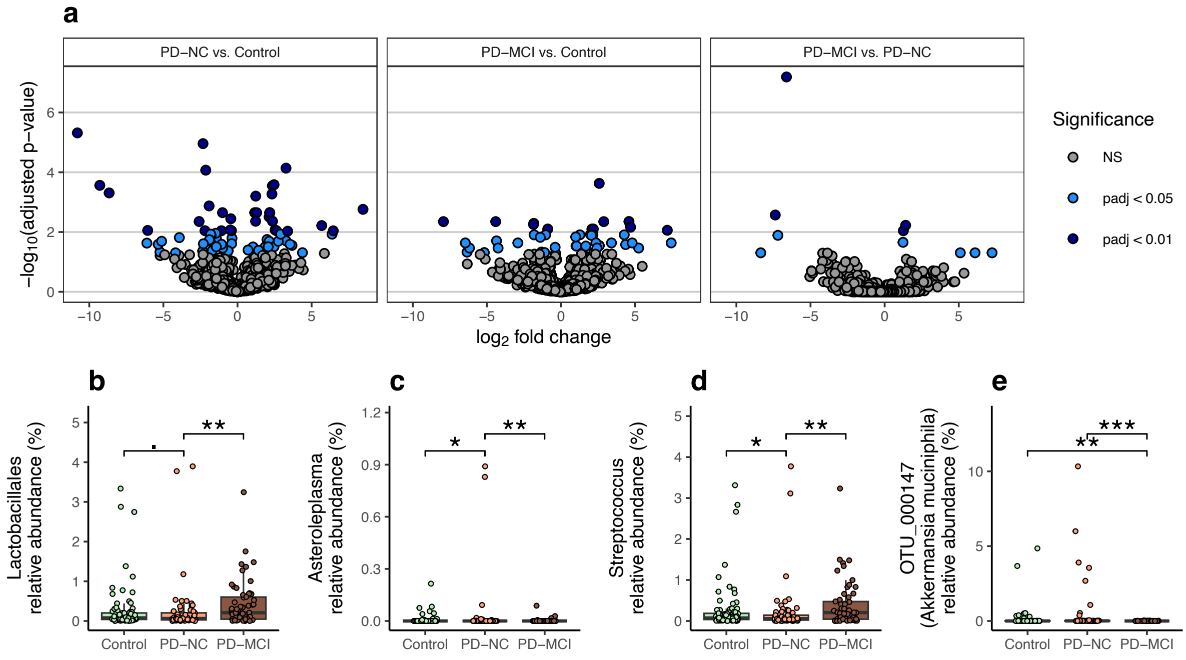

a. Volcano plots summarizing the results from differential abundance comparisons with DESeq2. **b-e.** Boxplots of the most significant differentially abundant taxa from DESeq2 comparisons of PD patients with and without MCI according to DESeq2 (multiple comparison corrected p-value < 0.01). Box hinges represent the 1st and 3rd quartiles, whiskers range from hinge to the highest and lowest values that are within 1.5*IQR of the hinge. Symbols: · : $0.1 > q > 0.05$; * : $q < 0.05$; ** : $q < 0.01$; *** : $q < 0.001$.

Supplementary Table 1: Alpha diversity results

Abbreviations: SCOPA-AUT: Scales for Outcomes in Parkinson's Disease-Autonomic questionnaire, MoCA: Montreal Cognitive Assessment, BMI: body mass index.

a. Single-variable comparisons

Binary categorical variables: Wilcoxon rank sum test, categorical variables with more than two categories: Kruskal-Wallis test, continuous numeric variables: Pearson correlations.

Variable	Index	Statistic	p	Test	r	ci
Sex	Chao1	3,993.000	**0.010**	Wilcoxon		
Caffeine intake (yes / no)	Chao1	1,167.000	0.111	Wilcoxon		
Years of education	Chao1	1.283	0.201	Pearson	0.089	-0.048, 0.222
Age	Chao1	1.225	0.222	Pearson	0.085	-0.052, 0.219
SCOPA-AUT Total Score	Chao1	1.176	0.241	Pearson	0.082	-0.056, 0.217
Antibiotics in last 6 months	Chao1	2,430.000	0.267	Wilcoxon		
Constipation (yes / no)	Chao1	3,995.000	0.307	Wilcoxon		
SCOPA-AUT gastrointestinal subscore	Chao1	1.000	0.318	Pearson	0.070	-0.068, 0.205
MoCA	Chao1	0.888	0.375	Pearson	0.062	-0.075, 0.196
BMI (categorical: obese or not)	Chao1	4,342.000	0.448	Wilcoxon		
Vegan or vegetarian diet	Chao1	820.000	0.459	Wilcoxon		
Education (categorical: <14 years / ≥ 14 years)	Chao1	5,079.000	0.483	Wilcoxon		
Diagnostic group (PD / Control)	Chao1	5,557.000	0.567	Wilcoxon		
Probiotics in last 6 months	Chao1	533.000	0.618	Wilcoxon		
BMI	Chao1	-0.494	0.622	Pearson	-0.034	-0.17, 0.102
Group (PD-MCI / PD-NC / Control)	Chao1	0.777	0.678	Kruskal		
Sex	InvSimpson	3,798.000	**0.002**	Wilcoxon		
Education (categorical: <14 years / ≥ 14 years)	InvSimpson	4,177.000	**0.005**	Wilcoxon		
Years of education	InvSimpson	2.504	**0.013**	Pearson	0.172	0.037, 0.301
Diagnostic group (PD / Control)	InvSimpson	5,924.000	0.154	Wilcoxon		
BMI (categorical: obese or not)	InvSimpson	4,485.000	0.254	Wilcoxon		
Antibiotics in last 6 months	InvSimpson	2,431.000	0.266	Wilcoxon		
Constipation (yes / no)	InvSimpson	3,993.000	0.304	Wilcoxon		
BMI	InvSimpson	-0.989	0.324	Pearson	-0.069	-0.203, 0.068
Vegan or vegetarian diet	InvSimpson	554.000	0.341	Wilcoxon		
Group (PD-MCI / PD-NC / Control)	InvSimpson	2.057	0.357	Kruskal		
Caffeine intake (yes / no)	InvSimpson	1,359.000	0.445	Wilcoxon		
Probiotics in last 6 months	InvSimpson	497.000	0.455	Wilcoxon		
MoCA	InvSimpson	0.716	0.475	Pearson	0.050	-0.087, 0.185

Variable	Index	Statistic	p	Test	r	ci
SCOPA-AUT Total Score	InvSimpson	0.649	0.517	Pearson	0.046	-0.092, 0.182
Age	InvSimpson	0.188	0.851	Pearson	0.013	-0.123, 0.149
SCOPA-AUT gastrointestinal subscore	InvSimpson	-0.057	0.955	Pearson	-0.004	-0.141, 0.133
Sex	Observed	3,989.000	**0.010**	Wilcoxon		
Caffeine intake (yes / no)	Observed	1,123.500	**0.075**	Wilcoxon		
SCOPA-AUT Total Score	Observed	1.530	0.128	Pearson	0.107	-0.031, 0.241
Constipation (yes / no)	Observed	3,824.500	0.145	Wilcoxon		
Years of education	Observed	1.339	0.182	Pearson	0.093	-0.044, 0.226
Antibiotics in last 6 months	Observed	2,446.000	0.243	Wilcoxon		
SCOPA-AUT gastrointestinal subscore	Observed	1.152	0.251	Pearson	0.080	-0.057, 0.215
Age	Observed	1.080	0.281	Pearson	0.075	-0.062, 0.209
Education (categorical: <14 years / ≥ 14 years)	Observed	4,931.000	0.297	Wilcoxon		
BMI (categorical: obese or not)	Observed	4,409.000	0.348	Wilcoxon		
MoCA	Observed	0.850	0.396	Pearson	0.059	-0.078, 0.194
Probiotics in last 6 months	Observed	491.500	0.433	Wilcoxon		
Diagnostic group (PD / Control)	Observed	5,620.000	0.472	Wilcoxon		
Group (PD-MCI / PD-NC / Control)	Observed	1.321	0.517	Kruskal		
BMI	Observed	-0.615	0.539	Pearson	-0.043	-0.178, 0.094
Vegan or vegetarian diet	Observed	782.000	0.618	Wilcoxon		
Sex	Shannon	3,580.000	**0.000**	Wilcoxon		
Education (categorical: <14 years / ≥ 14 years)	Shannon	4,331.000	**0.015**	Wilcoxon		
Years of education	Shannon	2.333	**0.021**	Pearson	0.160	0.025, 0.29
Antibiotics in last 6 months	Shannon	2,485.000	0.190	Wilcoxon		
Probiotics in last 6 months	Shannon	420.000	0.202	Wilcoxon		
Constipation (yes / no)	Shannon	3,901.000	0.207	Wilcoxon		
BMI (categorical: obese or not)	Shannon	4,480.000	0.260	Wilcoxon		
Diagnostic group (PD / Control)	Shannon	5,757.000	0.299	Wilcoxon		
Caffeine intake (yes / no)	Shannon	1,297.000	0.303	Wilcoxon		
SCOPA-AUT Total Score	Shannon	0.904	0.367	Pearson	0.064	-0.075, 0.199
Age	Shannon	0.893	0.373	Pearson	0.062	-0.075, 0.196
Group (PD-MCI / PD-NC / Control)	Shannon	1.312	0.519	Kruskal		
MoCA	Shannon	0.588	0.557	Pearson	0.041	-0.096, 0.176
BMI	Shannon	-0.566	0.572	Pearson	-0.039	-0.174, 0.097
Vegan or vegetarian diet	Shannon	640.000	0.687	Wilcoxon		
SCOPA-AUT gastrointestinal subscore	Shannon	0.015	0.988	Pearson	0.001	-0.136, 0.138

b. Linear model for inverse Simpson diversity, main grouping variable and confounders

**Predictor**	**Estimate**	**SE**	**t**	**p**
Intercept	23.537	1.961	12.002	**<0.001**
Group: PD without MCI	-3.547	1.966	-1.804	**0.073**
Group: PD with MCI	-4.050	2.101	-1.927	**0.055**
Sex	3.968	1.666	2.381	**0.018**
Age	1.120	1.603	0.699	0.485
BMI	-0.726	0.864	-0.840	0.402
Antibiotics in past 6 months	-3.425	2.440	-1.404	0.162
Education (categorical: <14 years / ≥ 14 years)	2.712	1.644	1.650	0.101
Constipation (yes / no)	4.572	1.856	2.464	**0.015**

c. Linear model for inverse Simpson diversity without control subjects

**Predictor**	**Estimate**	**SE**	**t**	**p**
Intercept	20.494	3.480	5.889	**<0.001**
MCI status	-0.531	2.176	-0.244	0.808
Sex	2.040	2.415	0.845	0.400
Age	1.877	2.196	0.855	0.395
BMI	-0.723	1.147	-0.631	0.530
Constipation (yes / no)	5.977	2.239	2.670	**0.009**
Education (categorical: <14 years / ≥ 14 years)	4.269	2.311	1.848	**0.067**
LEDD mg per day	-0.001	0.003	-0.207	0.836
Antibiotics in past 6 months	-3.580	3.590	-0.997	0.321
Disease duration since diagnosis years	-0.132	0.267	-0.495	0.622

Supplementary Table 2: Beta diversity results

Tables b-e show marginal effects.

a. PERMANOVA for individual variables

(model: distance matrix ~ variable)

Variable	Df	SumOfSqs	R2	F	p
SCOPA-AUT gastrointestinal subscore	1.000	0.763	0.012	2.473	**<0.001**
Group (PD-MCI / PD-NC / Control)	2.000	1.202	0.019	1.955	**<0.001**
Diagnostic group (PD / Control)	1.000	0.842	0.013	2.739	**<0.001**
Constipation (yes / no)	1.000	0.639	0.010	2.071	**<0.001**
BMI	1.000	0.570	0.009	1.845	**<0.001**
Sex	1.000	0.525	0.008	1.699	**0.002**
MoCA	1.000	0.482	0.008	1.560	**0.004**
BMI (categorical: obese or not)	1.000	0.457	0.007	1.476	**0.013**
SCOPA-AUT Total Score	1.000	0.451	0.007	1.455	**0.013**
Years of education	1.000	0.407	0.006	1.314	**0.049**
Age	1.000	0.379	0.006	1.225	0.104
Vegan or vegetarian diet	1.000	0.361	0.006	1.166	0.162
Education (categorical: <14 years / ≥ 14 years)	1.000	0.361	0.006	1.164	0.165
Antibiotics in last 6 months	1.000	0.306	0.005	0.988	0.484
Probiotics in last 6 months	1.000	0.278	0.004	0.897	0.707
Caffeine intake (yes / no)	1.000	0.247	0.004	0.796	0.895

b. PERMANOVA with confounders, all subjects

(model: distance matrix ~ Group + Sex + Age + BMI + Antibiotics + Constipation + Education)

term	df	SumOfSqs	R2	statistic	p
Group (PD-MCI / PD-NC / Control)	2.000	0.955	0.015	1.569	**<0.001**
Sex	1.000	0.495	0.008	1.628	**0.002**
Age	1.000	0.356	0.006	1.170	0.156
BMI	1.000	0.439	0.007	1.443	**0.016**
ATB in last 6 months	1.000	0.304	0.005	0.998	0.455
Constipation (yes / no)	1.000	0.432	0.007	1.420	**0.020**
Education (categorical: <14 years / ≥ 14 years)	1.000	0.297	0.005	0.976	0.516
Residual	199.000	60.546	0.943		
Total	207.000	64.189	1.000		

c. PERMANOVA with main confounders, Control vs PD-NC

(model: distance matrix ~ Group + Sex + BMI + Constipation)

term	df	SumOfSqs	R2	statistic	p
Group (Control / PD-NC)	1.000	0.633	0.014	2.114	**<0.001**
Sex	1.000	0.520	0.011	1.735	**0.002**
BMI	1.000	0.485	0.011	1.617	**0.003**
Constipation (yes / no)	1.000	0.323	0.007	1.076	0.303
Residual	145.000	43.446	0.954		
Total	149.000	45.544	1.000		

d. PERMANOVA with main confounders, Control vs PD-MCI

(model: distance matrix ~ Group + Sex + BMI + Constipation)

term	df	SumOfSqs	R2	statistic	p
Group (Control / PD-MCI)	1.000	0.424	0.009	1.405	**0.020**
Sex	1.000	0.408	0.009	1.352	**0.033**
BMI	1.000	0.364	0.008	1.207	0.115
Constipation (yes / no)	1.000	0.408	0.009	1.350	**0.034**
Residual	143.000	43.182	0.959		
Total	147.000	45.035	1.000		

e. PERMANOVA with main confounders, PD-NC vs PD-MCI

(model: distance matrix ~ MCI + Sex + BMI + Constipation + LEDD + Disease duration)

term	df	SumOfSqs	R2	statistic	p
MCI (yes / no)	1.000	0.343	0.009	1.092	0.272
Sex	1.000	0.303	0.008	0.965	0.536
BMI	1.000	0.494	0.013	1.574	**0.005**
Constipation (yes / no)	1.000	0.408	0.011	1.299	**0.061**
LEDD (mg / day)	1.000	0.321	0.009	1.023	0.410
Disease duration since diagnosis (years)	1.000	0.290	0.008	0.923	0.642
Residual	111.000	34.848	0.938		
Total	117.000	37.147	1.000		

f. ANOVA for group dispersions (betadisper)

Df	Sum Sq	Mean Sq	F value	Pr(>F)
2	0.020	0.010	4.546	0.012
205	0.441	0.002		

g. Pairwise comparisons between group dispersions (Tukey HSD)

Comparison	diff	lwr	upr	p adj
PD-NC vs. Control	0.018	-0.000	0.036	**0.058**
PD-MCI vs. Control	0.021	0.003	0.040	**0.020**
PD-MCI vs. PD-NC	0.003	-0.017	0.023	0.921

Supplementary Table 3: Detailed results for differential abundance comparisons

a. Comparisons of differential abundance tests with testDA for PD-only genus and ASV level data

AUC: Area Under Curve, FPR: False Positive Rate, FDR: False Discovery Rate. Asterisks indicate methods with equally good score considering the 90% confidence intervals.

Method label	Method name	AUC	FPR	FDR	Power	Score	Score (5%)	Score (95%)	
**Genus**									
lia	LIMMA - ALR	0.910	0.020	0.000	0.330	0.140	-1.090	0.350	*
lic	LIMMA - CLR	0.900	0.070	0.160	0.530	0.050	-0.510	0.220	*
lli2	Log LIMMA 2	0.890	0.070	0.190	0.530	0.010	-0.890	0.280	*
lli	Log LIMMA	0.850	0.060	0.140	0.400	-0.000	-0.340	0.200	*
qpo	Quasi-Poisson GLM	0.900	0.130	0.290	0.670	-0.030	-0.840	0.240	*
erq	EdgeR qll - TMM	0.920	0.120	0.400	0.730	-0.100	-0.430	0.120	*
vli	LIMMA voom	0.840	0.090	0.250	0.430	-0.100	-0.570	0.150	*
ds2	DESeq2 man. geoMeans	0.880	0.120	0.370	0.670	-0.120	-0.420	0.160	*
erq2	EdgeR qll - RLE	0.910	0.140	0.450	0.730	-0.140	-0.480	0.120	*
ds2x	DESeq2	0.870	0.130	0.450	0.670	-0.200	-0.540	0.200	*
zig	MgSeq ZIG	0.950	0.260	0.670	0.930	-0.250	-0.460	-0.120	*
znb	ZI-NegBin GLM	0.860	0.180	0.600	0.770	-0.330	-0.670	-0.070	*
neb	Negbinom GLM	0.810	0.170	0.560	0.670	-0.350	-0.780	-0.060	*
poi	Poisson GLM	0.820	0.740	0.870	1.000	-0.550	-0.710	-0.480	*
zpo	ZI-Poisson GLM	0.780	0.670	0.870	0.930	-0.610	-0.750	-0.490	*
**ASV**									
lia	LIMMA - ALR	0.870	0.050	0.000	0.260	0.100	-0.500	0.170	*
lli	Log LIMMA	0.830	0.050	0.000	0.090	0.030	-0.310	0.070	*
lic	LIMMA - CLR	0.890	0.050	0.090	0.300	0.030	-0.180	0.130	*
lli2	Log LIMMA 2	0.890	0.060	0.100	0.260	0.000	-0.450	0.150	*
ds2	DESeq2 man. geoMeans	0.920	0.090	0.390	0.560	-0.160	-0.430	0.080	*
qpo	Quasi-Poisson GLM	0.910	0.120	0.330	0.410	-0.170	-0.500	0.120	*
erq	EdgeR qll - TMM	0.910	0.100	0.400	0.520	-0.190	-0.480	0.010	*
vli	LIMMA voom	0.790	0.070	0.310	0.110	-0.280	-1.000	0.050	*
zig	MgSeq ZIG	0.950	0.200	0.680	0.890	-0.280	-0.440	-0.090	*
znb	ZI-NegBin GLM	0.900	0.150	0.620	0.810	-0.300	-0.510	-0.080	*
neb	Negbinom GLM	0.790	0.160	0.690	0.590	-0.520	-0.760	-0.360	
zpo	ZI-Poisson GLM	0.870	0.720	0.920	1.000	-0.550	-0.630	-0.500	
poi	Poisson GLM	0.860	0.740	0.920	1.000	-0.560	-0.650	-0.510	

b. Results from DAtest: DA.lic (showing taxa with multiple comparison corrected *p* < 0.1)

lfc: log2 fold change, AveExpr: average expression across all samples, t: t-statistic, B: B-statistic, padj: multiple comparison corrected *p*-value

Taxon	lfc	Ave Expr	t	B	p	padj	Contrast
**Phylum**							
Firmicutes	-0.323	5.040	-3.550	-0.334	0.000	0.005	PD-NC vs. Control
**Class**							
Clostridia	-0.300	5.576	-3.335	-0.960	0.001	0.017	PD-NC vs. Control
**Order**							
Clostridiales	-0.857	-1.016	-3.211	-1.272	0.002	0.031	PD-NC vs. Control
Lachnospirales	-0.332	5.055	-3.174	-1.378	0.002	0.031	PD-NC vs. Control
**Family**							
Butyricicoccaceae	-0.703	1.054	-3.255	-1.133	0.001	0.045	PD-MCI vs. Control
Lachnospiraceae	-0.323	5.304	-3.206	-1.274	0.002	0.045	PD-MCI vs. Control
Erysipelatoclostridiaceae	-0.656	0.835	-2.889	-2.143	0.004	0.081	PD-MCI vs. Control
Lachnospiraceae	-0.331	5.304	-3.512	-0.420	0.001	0.018	PD-NC vs. Control
Ruminococcaceae	-0.395	5.098	-3.467	-0.563	0.001	0.018	PD-NC vs. Control
Clostridiaceae	-0.855	-0.765	-3.318	-1.022	0.001	0.020	PD-NC vs. Control
Hydrogenoanaerobacterium	0.526	-1.722	2.976	-2.007	0.003	0.047	PD-NC vs. Control
Enterobacteriaceae	1.260	1.011	2.854	-2.336	0.005	0.054	PD-NC vs. Control
**Genus**							
Butyricicoccus	-0.772	1.483	-3.340	-0.878	0.001	0.069	PD-MCI vs. Control
DTU089	0.571	-0.762	3.221	-1.201	0.001	0.069	PD-MCI vs. Control
Hungatella	0.790	-0.751	3.091	-1.544	0.002	0.069	PD-MCI vs. Control
Lachnospiraceae NK4A136 group	-0.870	3.427	-3.087	-1.554	0.002	0.069	PD-MCI vs. Control
V9D2013 group	0.372	-1.451	3.299	-0.991	0.001	0.069	PD-MCI vs. Control
Acidaminococcus	0.997	-0.982	3.019	-1.728	0.003	0.071	PD-MCI vs. Control
[Eubacterium] eligens group	-1.040	1.996	-2.905	-2.010	0.004	0.085	PD-MCI vs. Control
Lachnospiraceae UCG 004	-0.841	1.434	-2.870	-2.095	0.005	0.085	PD-MCI vs. Control
DTU089	0.693	-0.762	4.181	1.896	0.000	0.006	PD-NC vs. Control
Agathobacter	-1.152	2.402	-3.786	0.516	0.000	0.015	PD-NC vs. Control
Anaerotruncus	0.670	-0.203	3.425	-0.643	0.001	0.037	PD-NC vs. Control
Lachnospiraceae UCG 004	-0.892	1.434	-3.255	-1.153	0.001	0.038	PD-NC vs. Control
Selenomonadales bacterium Marseille P2399	0.584	-1.087	3.188	-1.347	0.002	0.038	PD-NC vs. Control
UBA1819	0.758	0.615	3.298	-1.025	0.001	0.038	PD-NC vs. Control
UCG 003	-1.024	1.634	-3.165	-1.411	0.002	0.038	PD-NC vs. Control
Anaerofilum	0.306	-1.423	3.051	-1.733	0.003	0.044	PD-NC vs. Control
Clostridium sensu stricto 1	-0.797	-0.131	-3.046	-1.747	0.003	0.044	PD-NC vs. Control

Taxon	lfc	Ave Expr	t	B	p	padj	Contrast
[Eubacterium] eligens group	-0.989	1.996	-2.952	-2.003	0.004	0.047	PD-NC vs. Control
Hungatella	0.703	-0.751	2.940	-2.034	0.004	0.047	PD-NC vs. Control
Roseburia	-0.651	3.273	-2.927	-2.070	0.004	0.047	PD-NC vs. Control
Klebsiella	0.831	-1.207	2.852	-2.267	0.005	0.055	PD-NC vs. Control
Lachnospiraceae NK4A136 group	-0.738	3.427	-2.799	-2.404	0.006	0.060	PD-NC vs. Control
Faecalibacterium	-0.607	4.758	-2.705	-2.640	0.007	0.074	PD-NC vs. Control
Eisenbergiella	0.586	-0.839	2.593	-2.911	0.010	0.095	PD-NC vs. Control
Lachnospiraceae FCS020 group	-0.426	-0.601	-2.571	-2.965	0.011	0.095	PD-NC vs. Control
**ASV**							
OTU_000149 (Lachnospiraceae)	-1.186	1.606	-4.354	2.455	0.000	0.025	PD-NC vs. Control
OTU_000233 (genus UCG 005)	-1.011	0.966	-4.087	1.512	0.000	0.038	PD-NC vs. Control
OTU_000415 ([Eubacterium] coprostanoligenes group)	0.765	-0.213	3.916	0.936	0.000	0.049	PD-NC vs. Control
OTU_000070 (genus Roseburia)	-1.227	1.777	-3.702	0.245	0.000	0.073	PD-NC vs. Control
OTU_000132 (Lachnospiraceae UCG 004)	-0.907	1.829	-3.660	0.113	0.000	0.073	PD-NC vs. Control
OTU_003864 (Escherichia coli)	0.167	-0.474	3.621	-0.007	0.000	0.073	PD-NC vs. Control
OTU_000061 (genus Roseburia)	-1.176	1.839	-3.535	-0.269	0.001	0.086	PD-NC vs. Control

c. Results from DESeq2 (showing taxa with multiple comparison corrected *p* < 0.1)

baseMean: mean abundance across all data, lfc: log2 fold change, se: standard error for log2 fold change, stat: Wald statistic (lfc / se), padj: multiple comparison corrected *p*-value

Taxon	base Mean	lfc	se	stat	p	padj	Contrast
**Phylum**							
Euryarchaeota	5.600	2.246	0.796	2.822	0.005	0.048	PD-MCI vs. Control
Actinobacteriota	109.693	0.679	0.334	2.034	0.042	0.084	PD-MCI vs. Control
Bacteroidota	7,952.928	-0.296	0.135	-2.199	0.028	0.084	PD-MCI vs. Control
Firmicutes	8,639.306	-0.307	0.135	-2.284	0.022	0.084	PD-MCI vs. Control
Verrucomicrobiota	391.872	0.907	0.446	2.032	0.042	0.084	PD-MCI vs. Control
Firmicutes	8,639.306	-0.449	0.126	-3.568	0.000	0.004	PD-NC vs. Control
Bacteroidota	7,952.928	-0.371	0.126	-2.946	0.003	0.016	PD-NC vs. Control
Euryarchaeota	5.600	1.861	0.746	2.495	0.013	0.032	PD-NC vs. Control
Verrucomicrobiota	391.872	1.046	0.418	2.504	0.012	0.032	PD-NC vs. Control
**Class**							
Bacteroidia	8,117.731	-0.385	0.145	-2.663	0.008	0.052	PD-MCI vs. Control
Clostridia	7,995.758	-0.361	0.139	-2.604	0.009	0.052	PD-MCI vs. Control
Methanobacteria	6.001	2.244	0.802	2.799	0.005	0.052	PD-MCI vs. Control
Clostridia	7,995.758	-0.393	0.130	-3.028	0.002	0.025	PD-NC vs. Control
Methanobacteria	6.001	2.236	0.751	2.979	0.003	0.025	PD-NC vs. Control
**Order**							
Clostridiales	18.876	-1.838	0.483	-3.804	0.000	0.005	PD-MCI vs. Control
Enterobacterales	608.728	1.983	0.583	3.401	0.001	0.012	PD-MCI vs. Control
Lachnospirales	2,541.833	-0.402	0.149	-2.687	0.007	0.065	PD-MCI vs. Control
Methanobacteriales	6.458	2.201	0.801	2.748	0.006	0.065	PD-MCI vs. Control
Christensenellales	364.629	0.860	0.349	2.465	0.014	0.084	PD-MCI vs. Control
Izemoplasmatales	36.306	2.023	0.823	2.458	0.014	0.084	PD-MCI vs. Control
Lactobacillales	48.113	1.261	0.345	3.661	0.000	0.009	PD-MCI vs. PD-NC
Clostridiales	18.876	-2.326	0.454	-5.120	0.000	0.000	PD-NC vs. Control
Enterobacterales	608.728	2.355	0.545	4.318	0.000	0.000	PD-NC vs. Control
Methanobacteriales	6.458	2.263	0.751	3.014	0.003	0.031	PD-NC vs. Control
Verrucomicrobiales	344.216	1.358	0.520	2.611	0.009	0.081	PD-NC vs. Control
Pasteurellales	9.906	-1.591	0.641	-2.484	0.013	0.093	PD-NC vs. Control
Coriobacteriales	38.284	0.764	0.334	2.285	0.022	0.097	PD-NC vs. Control
Lachnospirales	2,541.833	-0.323	0.140	-2.310	0.021	0.097	PD-NC vs. Control
Lactobacillales	48.113	-0.766	0.333	-2.304	0.021	0.097	PD-NC vs. Control
Synergistales	0.866	2.794	1.239	2.255	0.024	0.097	PD-NC vs. Control
**Family**							
Clostridiaceae	17.827	-1.861	0.478	-3.896	0.000	0.006	PD-MCI vs. Control
Butyricicoccaceae	51.603	-0.896	0.260	-3.452	0.001	0.008	PD-MCI vs. Control
Enterobacteriaceae	546.862	2.084	0.599	3.480	0.001	0.008	PD-MCI vs. Control
Hydrogenoanaero-bacterium	4.245	2.175	0.610	3.563	0.000	0.008	PD-MCI vs. Control
Lachnospiraceae	2,475.689	-0.403	0.135	-2.985	0.003	0.032	PD-MCI vs. Control

Taxon	base Mean	lfc	se	stat	p	padj	Contrast
Methanobacteriaceae	7.366	2.137	0.814	2.624	0.009	0.083	PD-MCI vs. Control
Lactobacillaceae	2.003	2.654	1.059	2.506	0.012	0.089	PD-MCI vs. Control
Prevotellaceae	535.031	-1.821	0.729	-2.498	0.012	0.089	PD-MCI vs. Control
Christensenellaceae	378.914	0.853	0.353	2.414	0.016	0.094	PD-MCI vs. Control
Erysipelatoclostridiaceae	46.152	-0.697	0.291	-2.397	0.017	0.094	PD-MCI vs. Control
Streptococcaceae	46.152	1.241	0.350	3.548	0.000	0.022	PD-MCI vs. PD-NC
Clostridiaceae	17.827	-2.141	0.448	-4.782	0.000	0.000	PD-NC vs. Control
Enterobacteriaceae	546.862	2.468	0.560	4.404	0.000	0.000	PD-NC vs. Control
Hydrogenoanaero-bacterium	4.245	2.135	0.572	3.731	0.000	0.003	PD-NC vs. Control
Methanobacteriaceae	7.366	2.537	0.762	3.329	0.001	0.009	PD-NC vs. Control
Ruminococcaceae	2,086.726	-0.485	0.145	-3.346	0.001	0.009	PD-NC vs. Control
Lachnospiraceae	2,475.689	-0.406	0.126	-3.221	0.001	0.009	PD-NC vs. Control
Prevotellaceae	535.031	-2.196	0.682	-3.219	0.001	0.009	PD-NC vs. Control
Butyricicoccaceae	51.603	-0.640	0.242	-2.641	0.008	0.042	PD-NC vs. Control
Pasteurellaceae	10.971	-1.708	0.651	-2.626	0.009	0.042	PD-NC vs. Control
Streptococcaceae	46.152	-0.901	0.337	-2.672	0.008	0.042	PD-NC vs. Control
Akkermansiaceae	349.833	1.325	0.521	2.542	0.011	0.049	PD-NC vs. Control
Synergistaceae	1.104	3.167	1.275	2.483	0.013	0.053	PD-NC vs. Control
Clostridium sp. CAG:306	3.522	-3.233	1.333	-2.425	0.015	0.058	PD-NC vs. Control
Coriobacteriaceae	18.767	1.102	0.467	2.360	0.018	0.064	PD-NC vs. Control
**Genus**							
Hungatella	8.740	2.575	0.540	4.770	0.000	0.000	PD-MCI vs. Control
Butyricicoccus	48.730	-1.038	0.295	-3.520	0.000	0.012	PD-MCI vs. Control
Clostridium sensu stricto 1	19.099	-1.818	0.492	-3.693	0.000	0.012	PD-MCI vs. Control
DTU089	4.262	1.268	0.364	3.486	0.000	0.012	PD-MCI vs. Control
Escherichia Shigella	397.404	2.148	0.601	3.573	0.000	0.012	PD-MCI vs. Control
Faecalitalea	2.018	4.263	1.239	3.440	0.001	0.012	PD-MCI vs. Control
Selenomonadales bacterium Marseille P2399	4.047	2.050	0.606	3.386	0.001	0.013	PD-MCI vs. Control
UBA1819	21.998	0.990	0.298	3.316	0.001	0.015	PD-MCI vs. Control
Methanobrevibacter	8.945	2.583	0.836	3.090	0.002	0.028	PD-MCI vs. Control
Bilophila	57.653	1.010	0.336	3.009	0.003	0.029	PD-MCI vs. Control
Flavonifractor	21.444	1.046	0.349	2.994	0.003	0.029	PD-MCI vs. Control
Veillonella	25.239	1.672	0.552	3.028	0.002	0.029	PD-MCI vs. Control
UCG 009	3.041	1.438	0.513	2.805	0.005	0.049	PD-MCI vs. Control
V9D2013 group	1.045	1.482	0.568	2.609	0.009	0.083	PD-MCI vs. Control
Asteroleplasma	2.230	-7.379	1.721	-4.288	0.000	0.003	PD-MCI vs. PD-NC
Streptococcus	48.660	1.417	0.359	3.944	0.000	0.006	PD-MCI vs. PD-NC
Cloacibacillus	1.025	-3.919	1.255	-3.123	0.002	0.089	PD-MCI vs. PD-NC
Hungatella	8.740	2.324	0.505	4.603	0.000	0.001	PD-NC vs. Control
UBA1819	21.998	1.233	0.279	4.421	0.000	0.001	PD-NC vs. Control
Clostridium sensu stricto 1	19.099	-1.915	0.460	-4.163	0.000	0.001	PD-NC vs. Control
Anaerotruncus	8.935	1.162	0.302	3.841	0.000	0.002	PD-NC vs. Control
DTU089	4.262	1.292	0.339	3.807	0.000	0.002	PD-NC vs. Control

Taxon	base Mean	lfc	se	stat	p	padj	Contrast
Escherichia Shigella	397.404	2.177	0.562	3.872	0.000	0.002	PD-NC vs. Control
Roseburia	280.730	-1.012	0.260	-3.893	0.000	0.002	PD-NC vs. Control
Selenomonadales bacterium Marseille P2399	4.047	2.174	0.566	3.838	0.000	0.002	PD-NC vs. Control
Klebsiella	63.805	5.683	1.613	3.523	0.000	0.006	PD-NC vs. Control
Lachnospiraceae UCG 004	54.618	-1.084	0.323	-3.361	0.001	0.009	PD-NC vs. Control
Methanobrevibacter	8.945	2.630	0.782	3.362	0.001	0.009	PD-NC vs. Control
Romboutsia	10.179	-1.473	0.449	-3.281	0.001	0.011	PD-NC vs. Control
[Ruminococcus] gauvreauii group	6.537	-1.423	0.453	-3.142	0.002	0.017	PD-NC vs. Control
Streptococcus	48.660	-1.004	0.346	-2.899	0.004	0.034	PD-NC vs. Control
Oscillibacter	87.476	0.711	0.252	2.828	0.005	0.040	PD-NC vs. Control
Eisenbergiella	6.614	1.424	0.509	2.800	0.005	0.041	PD-NC vs. Control
Akkermansia	361.668	1.446	0.526	2.747	0.006	0.043	PD-NC vs. Control
Faecalibacterium	1,042.063	-0.630	0.230	-2.740	0.006	0.043	PD-NC vs. Control
Prevotella	376.814	-2.347	0.861	-2.727	0.006	0.043	PD-NC vs. Control
Asteroleplasma	2.230	4.383	1.644	2.666	0.008	0.049	PD-NC vs. Control
Collinsella	19.987	1.193	0.473	2.524	0.012	0.071	PD-NC vs. Control
Cloacibacillus	1.025	3.002	1.208	2.486	0.013	0.075	PD-NC vs. Control
Butyricicoccus	48.730	-0.666	0.275	-2.419	0.016	0.087	PD-NC vs. Control
Lachnospiraceae UCG 001	39.419	-1.094	0.458	-2.386	0.017	0.091	PD-NC vs. Control
Blautia	122.376	-0.465	0.197	-2.360	0.018	0.091	PD-NC vs. Control
Lachnospiraceae NK4A136 group	399.884	-0.713	0.303	-2.355	0.019	0.091	PD-NC vs. Control
**ASV**							
OTU_000126 (Escherichia Shigella)	37.211	4.581	1.010	4.536	0.000	0.004	PD-MCI vs. Control
OTU_000147 (Akkermansia muciniphila)	33.784	-4.410	1.042	-4.231	0.000	0.004	PD-MCI vs. Control
OTU_000519 (genus Hungatella)	5.527	2.882	0.674	4.278	0.000	0.004	PD-MCI vs. Control
OTU_000858 (family unclassified)	2.681	-7.934	1.840	-4.313	0.000	0.004	PD-MCI vs. Control
OTU_000546 (Bacteroides dorei)	9.301	4.692	1.149	4.083	0.000	0.007	PD-MCI vs. Control
OTU_000415 (family [Eubacterium] coprostanoligenes group)	5.880	7.166	1.799	3.984	0.000	0.009	PD-MCI vs. Control
OTU_000220 (genus Butyricicoccus)	16.697	-1.406	0.368	-3.815	0.000	0.015	PD-MCI vs. Control
OTU_000756 (genus Romboutsia)	2.249	-4.353	1.172	-3.713	0.000	0.020	PD-MCI vs. Control
OTU_000324 (Klebsiella pneumoniae)	21.280	7.428	2.069	3.590	0.000	0.023	PD-MCI vs. Control

Taxon	base Mean	lfc	se	stat	p	padj	Contrast
OTU_000345 (genus Veillonella)	7.399	2.353	0.661	3.558	0.000	0.023	PD-MCI vs. Control
OTU_000346 (genus UCG 005)	24.644	-6.460	1.823	-3.544	0.000	0.023	PD-MCI vs. Control
OTU_000986 (genus UCG 003)	2.555	-2.914	0.826	-3.528	0.000	0.023	PD-MCI vs. Control
OTU_001025 (genus Bifidobacterium)	2.033	3.402	0.945	3.602	0.000	0.023	PD-MCI vs. Control
OTU_002026 (genus Christensenellaceae R 7 group)	1.232	4.796	1.327	3.613	0.000	0.023	PD-MCI vs. Control
OTU_001190 (Faecalitalea cylindroides)	2.337	4.354	1.253	3.475	0.001	0.026	PD-MCI vs. Control
OTU_000284 (genus Barnesiella)	15.739	-6.178	1.851	-3.338	0.001	0.034	PD-MCI vs. Control
OTU_000562 (genus Hungatella)	3.279	2.421	0.719	3.366	0.001	0.034	PD-MCI vs. Control
OTU_000679 (genus Faecalibacterium)	1.527	5.236	1.568	3.340	0.001	0.034	PD-MCI vs. Control
OTU_001514 (genus Agathobacter)	1.107	-4.249	1.272	-3.339	0.001	0.034	PD-MCI vs. Control
OTU_000087 (Roseburia hominis A2 183)	40.936	-1.326	0.412	-3.221	0.001	0.047	PD-MCI vs. Control
OTU_000236 (genus Prevotella)	16.878	-6.343	1.966	-3.226	0.001	0.047	PD-MCI vs. Control
OTU_000132 (genus Lachnospiraceae UCG 004)	23.731	-1.109	0.348	-3.186	0.001	0.049	PD-MCI vs. Control
OTU_000656 (genus UCG 002)	4.508	-5.179	1.626	-3.185	0.001	0.049	PD-MCI vs. Control
OTU_000092 (Bacteroides ovatus)	31.882	2.178	0.693	3.145	0.002	0.054	PD-MCI vs. Control
OTU_000139 (family unclassified)	33.451	3.097	1.004	3.084	0.002	0.055	PD-MCI vs. Control
OTU_000228 (genus Clostridium sensu stricto 1)	11.251	-1.789	0.575	-3.111	0.002	0.055	PD-MCI vs. Control
OTU_000258 (genus Flavonifractor)	11.075	1.370	0.445	3.081	0.002	0.055	PD-MCI vs. Control
OTU_000567 (genus Parabacteroides)	5.937	3.429	1.111	3.085	0.002	0.055	PD-MCI vs. Control
OTU_002219 (family unclassified)	0.852	2.323	0.754	3.081	0.002	0.055	PD-MCI vs. Control
OTU_000977 (family Lachnospiraceae)	2.073	-1.451	0.473	-3.069	0.002	0.055	PD-MCI vs. Control
OTU_001105 (genus Butyricicoccus)	1.590	-2.174	0.713	-3.048	0.002	0.056	PD-MCI vs. Control
OTU_001171 (family Ruminococcaceae)	2.246	-5.462	1.794	-3.045	0.002	0.056	PD-MCI vs. Control

Taxon	base Mean	lfc	se	stat	p	padj	Contrast
OTU_000633 (Bacteroides vulgatus)	2.340	-1.864	0.629	-2.964	0.003	0.071	PD-MCI vs. Control
OTU_000771 (genus Selenomonadales bacterium Marseille P2399)	3.965	2.023	0.691	2.928	0.003	0.078	PD-MCI vs. Control
OTU_000486 (Methanobrevibacter smithii)	10.201	2.488	0.859	2.897	0.004	0.081	PD-MCI vs. Control
OTU_000676 (genus UCG 002)	8.809	-5.115	1.764	-2.899	0.004	0.081	PD-MCI vs. Control
OTU_001208 (Anaerotruncus sp. MT15)	1.816	0.963	0.336	2.870	0.004	0.086	PD-MCI vs. Control
OTU_000008 (Escherichia coli)	337.823	1.720	0.603	2.852	0.004	0.087	PD-MCI vs. Control
OTU_000159 (genus UBA1819)	22.810	0.909	0.320	2.842	0.004	0.087	PD-MCI vs. Control
OTU_000961 (genus Agathobacter)	2.065	-1.391	0.489	-2.844	0.004	0.087	PD-MCI vs. Control
OTU_000233 (genus UCG 005)	13.244	-1.446	0.513	-2.821	0.005	0.090	PD-MCI vs. Control
OTU_000147 (Akkermansia muciniphila)	33.784	-6.620	1.010	-6.558	0.000	0.000	PD-MCI vs. PD-NC
OTU_001484 (genus Asteroleplasma)	2.029	-7.200	1.696	-4.246	0.000	0.013	PD-MCI vs. PD-NC
OTU_000059 (genus Prevotella)	26.177	5.104	1.392	3.667	0.000	0.050	PD-MCI vs. PD-NC
OTU_000067 (genus Prevotellaceae NK3B31 group)	43.262	-8.355	2.282	-3.662	0.000	0.050	PD-MCI vs. PD-NC
OTU_000153 (Prevotella copri)	27.151	7.246	1.936	3.743	0.000	0.050	PD-MCI vs. PD-NC
OTU_000693 (Bacteroides nordii)	2.966	6.101	1.628	3.748	0.000	0.050	PD-MCI vs. PD-NC
OTU_001446 (genus Oscillospira)	1.606	-3.860	1.067	-3.617	0.000	0.051	PD-MCI vs. PD-NC
OTU_000696 (genus Faecalibacterium)	2.531	-4.176	1.167	-3.580	0.000	0.051	PD-MCI vs. PD-NC
OTU_000089 (family unclassified)	28.371	-3.542	1.014	-3.493	0.000	0.063	PD-MCI vs. PD-NC
OTU_000158 (Streptococcus salivarius)	21.292	1.460	0.430	3.397	0.001	0.081	PD-MCI vs. PD-NC
OTU_000058 (Bacteroides fragilis)	53.210	-2.594	0.789	-3.286	0.001	0.097	PD-MCI vs. PD-NC
OTU_000401 (Streptococcus parasanguinis)	7.430	1.815	0.550	3.297	0.001	0.097	PD-MCI vs. PD-NC

Taxon	base Mean	lfc	se	stat	p	padj	Contrast
OTU_000472 (Lachnospiraceae NK4A136 group)	5.411	-2.027	0.623	-3.253	0.001	0.097	PD-MCI vs. PD-NC
OTU_001455 (genus Cloacibacillus)	1.201	-4.191	1.283	-3.266	0.001	0.097	PD-MCI vs. PD-NC
OTU_000153 (Prevotella copri)	27.151	-10.801	1.862	-5.800	0.000	0.000	PD-NC vs. Control
OTU_000519 (genus Hungatella)	5.527	3.272	0.629	5.199	0.000	0.000	PD-NC vs. Control
OTU_000198 (genus Prevotella)	14.395	-9.289	1.909	-4.867	0.000	0.000	PD-NC vs. Control
OTU_000236 (genus Prevotella)	16.878	-8.657	1.845	-4.692	0.000	0.000	PD-NC vs. Control
OTU_000324 (Klebsiella pneumoniae)	21.280	8.466	1.934	4.378	0.000	0.002	PD-NC vs. Control
OTU_000159 (genus UBA1819)	22.810	1.225	0.299	4.096	0.000	0.004	PD-NC vs. Control
OTU_000206 (Lachnoclostridium YL32)	25.455	2.361	0.575	4.104	0.000	0.004	PD-NC vs. Control
OTU_000025 (Bacteroides vulgatus)	159.957	-2.589	0.638	-4.061	0.000	0.004	PD-NC vs. Control
OTU_000693 (Bacteroides nordii)	2.966	-6.056	1.565	-3.869	0.000	0.009	PD-NC vs. Control
OTU_000415 ([Eubacterium] coprostanoligenes group)	5.880	6.459	1.684	3.836	0.000	0.009	PD-NC vs. Control
OTU_001025 (genus Bifidobacterium)	2.033	3.385	0.890	3.805	0.000	0.009	PD-NC vs. Control
OTU_000070 (genus Roseburia)	61.921	-1.808	0.490	-3.686	0.000	0.012	PD-NC vs. Control
OTU_000285 (Klebsiella variicola)	24.485	6.378	1.726	3.696	0.000	0.012	PD-NC vs. Control
OTU_000715 (genus Oscillospira)	4.723	2.914	0.790	3.687	0.000	0.012	PD-NC vs. Control
OTU_000061 (genus Roseburia)	52.859	-1.779	0.488	-3.648	0.000	0.013	PD-NC vs. Control
OTU_000756 (genus Romboutsia)	2.249	-3.924	1.095	-3.584	0.000	0.015	PD-NC vs. Control
OTU_000008 (Escherichia coli)	337.823	1.991	0.564	3.530	0.000	0.018	PD-NC vs. Control
OTU_000401 (Streptococcus parasanguinis)	7.430	-1.890	0.539	-3.510	0.000	0.018	PD-NC vs. Control
OTU_000126 (genus Escherichia Shigella)	37.211	3.279	0.945	3.471	0.001	0.020	PD-NC vs. Control
OTU_000201 (Bacteroides thetaiotaomicron)	19.718	2.137	0.626	3.412	0.001	0.020	PD-NC vs. Control
OTU_000228 (genus Clostridium sensu	11.251	-1.843	0.537	-3.434	0.001	0.020	PD-NC vs. Control

Taxon	base Mean	lfc	se	stat	p	padj	Contrast
stricto 1)							
OTU_000486 (Methanobrevibacter smithii)	10.201	2.749	0.804	3.421	0.001	0.020	PD-NC vs. Control
OTU_001249 (genus CAG 56)	1.565	-5.117	1.498	-3.417	0.001	0.020	PD-NC vs. Control
OTU_000232 (family unclassified)	17.454	-6.113	1.819	-3.362	0.001	0.023	PD-NC vs. Control
OTU_000753 (genus Clostridium sensu stricto 1)	1.961	-2.301	0.690	-3.336	0.001	0.025	PD-NC vs. Control
OTU_000132 (genus Lachnospiraceae UCG 004)	23.731	-1.072	0.325	-3.296	0.001	0.026	PD-NC vs. Control
OTU_000546 (Bacteroides dorei)	9.301	3.542	1.076	3.292	0.001	0.026	PD-NC vs. Control
OTU_000604 (genus Lachnospira)	1.935	-5.349	1.620	-3.301	0.001	0.026	PD-NC vs. Control
OTU_000358 (genus Christensenellaceae R 7 group)	9.936	3.677	1.128	3.259	0.001	0.028	PD-NC vs. Control
OTU_000323 (genus NK4A214 group)	15.919	2.461	0.765	3.217	0.001	0.031	PD-NC vs. Control
OTU_000746 (genus Blautia)	3.079	-1.431	0.447	-3.200	0.001	0.031	PD-NC vs. Control
OTU_000771 (genus Selenomonadales bacterium Marseille P2399)	3.965	2.069	0.646	3.202	0.001	0.031	PD-NC vs. Control
OTU_000346 (genus UCG 005)	24.644	-5.232	1.702	-3.074	0.002	0.047	PD-NC vs. Control
OTU_000373 (Bacteroides stercoris ATCC 43183)	6.876	-4.179	1.372	-3.046	0.002	0.050	PD-NC vs. Control
OTU_000177 (family unclassified)	28.789	5.861	1.939	3.023	0.003	0.052	PD-NC vs. Control
OTU_001077 (family Lachnospiraceae)	2.005	-2.323	0.777	-2.989	0.003	0.056	PD-NC vs. Control
OTU_000676 (genus UCG 002)	8.809	-4.911	1.651	-2.974	0.003	0.058	PD-NC vs. Control
OTU_001301 (genus NK4A214 group)	1.214	2.308	0.781	2.957	0.003	0.059	PD-NC vs. Control
OTU_000059 (genus Prevotella)	26.177	-3.945	1.343	-2.938	0.003	0.061	PD-NC vs. Control
OTU_001037 (genus Intestinimonas)	2.163	1.694	0.577	2.935	0.003	0.061	PD-NC vs. Control
OTU_000283 ([Eubacterium] siraeum DSM 15702)	13.021	-5.218	1.785	-2.924	0.003	0.061	PD-NC vs. Control

Taxon	base Mean	lfc	se	stat	p	padj	Contrast
OTU_000149 (family Lachnospiraceae)	24.301	-1.212	0.427	-2.842	0.004	0.073	PD-NC vs. Control
OTU_000386 (Haemophilus parainfluenzae)	6.487	-1.932	0.675	-2.861	0.004	0.073	PD-NC vs. Control
OTU_000651 (genus Christensenellaceae R 7 group)	8.030	3.284	1.156	2.840	0.005	0.073	PD-NC vs. Control
OTU_001373 (genus Christensenellaceae R 7 group)	1.950	1.883	0.661	2.848	0.004	0.073	PD-NC vs. Control
OTU_000160 (genus Blautia)	26.782	-0.873	0.313	-2.788	0.005	0.080	PD-NC vs. Control
OTU_000327 (Dorea formicigenerans ATCC 27755)	8.919	-0.844	0.301	-2.799	0.005	0.080	PD-NC vs. Control
OTU_000656 (genus UCG 002)	4.508	-4.241	1.520	-2.790	0.005	0.080	PD-NC vs. Control
OTU_000089 (family unclassified)	28.371	2.687	0.972	2.763	0.006	0.084	PD-NC vs. Control
OTU_000092 (Bacteroides ovatus)	31.882	1.788	0.648	2.759	0.006	0.084	PD-NC vs. Control
OTU_000282 (genus UCG 003)	10.241	-1.366	0.498	-2.741	0.006	0.087	PD-NC vs. Control
OTU_000910 (genus UCG 005)	2.172	-1.164	0.427	-2.725	0.006	0.090	PD-NC vs. Control

d. Results from ANCOM-BC2 (showing taxa with multiple comparison corrected *p* < 0.1)

lfc: log fold change from ANCOM-BC2 log-linear (natural log) model, se: standard error of lfc, stat: W-statistic (lfc/se), padj: multiple comparison corrected *p*-value, test: type of test (primary for default test, pairwise for pairwise directional test)

Taxon	lfc	se	stat	p	padj	Contrast	Test
**Order**							
Lachnospirales	-0.364	0.118	-3.082	0.002	0.076	PD-MCI vs. Control	primary
Clostridiales	-0.851	0.279	-3.055	0.002	0.056	PD-NC vs. Control	primary
Lachnospirales	-0.327	0.110	-2.963	0.003	0.056	PD-NC vs. Control	primary
**Family**							
Butyricicoccaceae	-0.743	0.233	-3.196	0.001	0.047	PD-MCI vs. Control	primary
Lachnospiraceae	-0.363	0.108	-3.366	0.001	0.047	PD-MCI vs. Control	primary
Erysipelatoclostridiaceae	-0.697	0.241	-2.889	0.004	0.088	PD-MCI vs. Control	primary
Clostridiaceae	-0.851	0.275	-3.094	0.002	0.067	PD-NC vs. Control	primary
Lachnospiraceae	-0.327	0.101	-3.237	0.001	0.067	PD-NC vs. Control	primary
Enterobacteriaceae	1.264	0.448	2.819	0.005	0.082	PD-NC vs. Control	primary
Ruminococcaceae	-0.391	0.136	-2.877	0.004	0.082	PD-NC vs. Control	primary
**Genus**							
Agathobacter	-1.207	0.325	-3.709	0.000	0.038	PD-NC vs. Control	primary
DTU089	0.639	0.184	3.482	0.000	0.046	PD-NC vs. Control	primary
Lachnospiraceae UCG 004	-0.947	0.281	-3.365	0.001	0.047	PD-NC vs. Control	primary
Clostridium sensu stricto 1	-0.851	0.274	-3.106	0.002	0.070	PD-NC vs. Control	primary
UCG 003	-1.079	0.344	-3.137	0.002	0.070	PD-NC vs. Control	primary
[Eubacterium] eligens group	-1.043	0.359	-2.905	0.004	0.077	PD-NC vs. Control	primary
Anaerotruncus	0.616	0.209	2.951	0.003	0.077	PD-NC vs. Control	primary
Roseburia	-0.705	0.243	-2.901	0.004	0.077	PD-NC vs. Control	primary
UBA1819	0.704	0.234	3.008	0.003	0.077	PD-NC vs. Control	primary
Lachnospiraceae NK4A136 group	-0.792	0.281	-2.816	0.005	0.090	PD-NC vs. Control	primary
Faecalibacterium	-0.662	0.245	-2.699	0.007	0.099	PD-NC vs. Control	primary
Hungatella	0.650	0.240	2.709	0.007	0.099	PD-NC vs. Control	primary
Klebsiella	0.777	0.285	2.728	0.006	0.099	PD-NC vs. Control	primary
**ASV**							
OTU_000149 (family Lachnospiraceae)	-1.225	0.283	-4.332	0.000	0.018	PD-NC vs. Control	primary
OTU_000149 (family Lachnospiraceae)	-1.225	0.283	-4.332	0.000	0.026	PD-NC vs. Control	pairwise
OTU_000233 (genus UCG 005)	-1.044	0.263	-3.976	0.000	0.042	PD-NC vs. Control	primary
OTU_000070 (genus Roseburia)	-1.265	0.339	-3.732	0.000	0.051	PD-NC vs. Control	primary
OTU_000132 (genus Lachnospiraceae UCG 004)	-0.939	0.254	-3.701	0.000	0.051	PD-NC vs. Control	primary
OTU_000415 ([Eubacterium] coprostanoligenes group)	0.753	0.198	3.805	0.000	0.051	PD-NC vs. Control	primary
OTU_000061 (genus Roseburia)	-1.208	0.344	-3.515	0.000	0.087	PD-NC vs. Control	primary
