# Supplementary material for: Gut microbiome is not associated with mild cognitive impairment in Parkinson’s disease
Source: NPJ Parkinsons Dis. 2024 Apr 6;10:78. doi: 10.1038/s41531-024-00687-1 (PMC10998870; doi:10.1038/s41531-024-00687-1)
Supplement: Supplementary file 2 — Reporting Summary [file 41531_2024_687_MOESM2_ESM.pdf]

nature portfolio

Corresponding author(s): Velma T. E. Aho

Last updated by author(s): Mar 4, 2024

Reporting Summary

Nature Portfolio wishes to improve the reproducibility of the work that we publish. This form provides structure for consistency and transparency in reporting. For further information on Nature Portfolio policies, see our Editorial Policies and the Editorial Policy Checklist.

Statistics

For all statistical analyses, confirm that the following items are present in the figure legend, table legend, main text, or Methods section.

n/a	Confirmed	
☐	☒	The exact sample size (*n*) for each experimental group/condition, given as a discrete number and unit of measurement
☒	☐	A statement on whether measurements were taken from distinct samples or whether the same sample was measured repeatedly
☐	☒	The statistical test(s) used AND whether they are one- or two-sided<br>*Only common tests should be described solely by name; describe more complex techniques in the Methods section.*
☐	☒	A description of all covariates tested
☐	☒	A description of any assumptions or corrections, such as tests of normality and adjustment for multiple comparisons
☐	☒	A full description of the statistical parameters including central tendency (e.g. means) or other basic estimates (e.g. regression coefficient) AND variation (e.g. standard deviation) or associated estimates of uncertainty (e.g. confidence intervals)
☐	☒	For null hypothesis testing, the test statistic (e.g. *F*, *t*, *r*) with confidence intervals, effect sizes, degrees of freedom and *P* value noted<br>*Give P values as exact values whenever suitable.*
☒	☐	For Bayesian analysis, information on the choice of priors and Markov chain Monte Carlo settings
☒	☐	For hierarchical and complex designs, identification of the appropriate level for tests and full reporting of outcomes
☐	☒	Estimates of effect sizes (e.g. Cohen's *d*, Pearson's *r*), indicating how they were calculated

Our web collection on statistics for biologists contains articles on many of the points above.

Software and code

Policy information about availability of computer code

Data collection	No code was used for data collection.
Data analysis	The R code for this study is available at https://gitlab.lcsb.uni.lu/ESB/ncer-mci-microbiome.

For manuscripts utilizing custom algorithms or software that are central to the research but not yet described in published literature, software must be made available to editors and reviewers. We strongly encourage code deposition in a community repository (e.g. GitHub). See the Nature Portfolio guidelines for submitting code & software for further information.

Data

Policy information about availability of data

All manuscripts must include a data availability statement. This statement should provide the following information, where applicable:

- Accession codes, unique identifiers, or web links for publicly available datasets
- A description of any restrictions on data availability
- For clinical datasets or third party data, please ensure that the statement adheres to our policy

Patient data used in the preparation of this manuscript were obtained from the National Centre of Excellence in Research on Parkinson's Disease (NCER-PD). NCER-PD datasets are not publicly available, as they are linked to the Luxembourg Parkinson's Study and its internal regulations. The NCER-PD Consortium is willing to share its available data. Its access policy was devised based on the study ethics documents, including the informed consent form, as approved by the national ethics committee. Requests to access datasets should be directed to the Data and Sample Access Committee via email: request.ncer-pd@uni.lu.

Research involving human participants, their data, or biological material

Policy information about studies with human participants or human data. See also policy information about sex, gender (identity/presentation), and sexual orientation and race, ethnicity and racism.

Reporting on sex and gender	Sex is tested as a potential confounding variable in the analyses, but not discussed in any detail. This is done because of its relevance to the analyses (e.g. male sex is associated with higher risk of Parkinson's disease). We call this variable "sex" and not "gender" as we are interested in disease-relevant biological effects.
Reporting on race, ethnicity, or other socially relevant groupings	Data regarding ethnicity, race or other similar groupings is not presented.
Population characteristics	The study population consisted of 58 people with PD and MCI (PD-MCI), 60 people with PD without cognitive impairment (PD-NC), and 90 cognitively normal control subjects. In addition to comparisons between these three groups, we tested a number of potential confounding variables. All of the main tests were corrected for sex, age, BMI, antibiotic use, constipation and duration of education.
Recruitment	The subjects were not recruited for the present study, but for the Luxembourg Parkinson's Study. The details have been published in Hipp, G. et al. The Luxembourg Parkinson's Study: A comprehensive approach for stratification and early diagnosis. Front Aging Neurosci 10, 326 (2018).
Ethics oversight	The Luxembourg Parkinson's Study was conducted according to the Declaration of Helsinki, with approval from the National Ethics Board (CNER Ref: 201407/13) and Data Protection Committee (CNPD Ref: 446/2017).

Note that full information on the approval of the study protocol must also be provided in the manuscript.

Field-specific reporting

Please select the one below that is the best fit for your research. If you are not sure, read the appropriate sections before making your selection.

☒ Life sciences ☐ Behavioural & social sciences ☐ Ecological, evolutionary & environmental sciences

For a reference copy of the document with all sections, see nature.com/documents/nr-reporting-summary-flat.pdf

Life sciences study design

All studies must disclose on these points even when the disclosure is negative.

Sample size	As this is a study using pre-existing data, sample size was defined based on the availability of data from subjects that fit our selection criteria.
Data exclusions	At the start of our analyses, the data consisted of 468 individuals. Additional exclusions were implemented as follows:<br>1) 236 subjects due to age <= 64 years,<br>2) 3 subjects due to missing information on education or BMI,<br>3) 5 subjects due to having < 10,000 sequence reads left after removing rare ASVs (defined as those present in < 10% of samples) and any ASVs classified as chloroplasts or mitochondria,<br>4) 16 subjects due to reported use of corticosteroids or immunosuppressants in the past 6 months.<br>After these exclusions, the final data set used for analyses consisted of data from 208 individuals.
Replication	The manuscript in itself represents an attempt of replicating a previously published study. For internal replication, we included differential abundance comparisons with three different tests to see whether they agree.
Randomization	This is an observational case-control study, so randomization is not relevant. As described above, our tests were corrected for potential confounding effects of age, sex, BMI, antibiotic use, constipation and duration of education.
Blinding	This is an observational case-control study, so blinding is not relevant.

Reporting for specific materials, systems and methods

We require information from authors about some types of materials, experimental systems and methods used in many studies. Here, indicate whether each material, system or method listed is relevant to your study. If you are not sure if a list item applies to your research, read the appropriate section before selecting a response.

Materials & experimental systems

n/a	Involved in the study
☒	☐ Antibodies
☒	☐ Eukaryotic cell lines
☒	☐ Palaeontology and archaeology
☒	☐ Animals and other organisms
☒	☐ Clinical data
☒	☐ Dual use research of concern
☒	☐ Plants

Methods

n/a	Involved in the study
☒	☐ ChIP-seq
☒	☐ Flow cytometry
☒	☐ MRI-based neuroimaging

Plants

Seed stocks	*Report on the source of all seed stocks or other plant material used. If applicable, state the seed stock centre and catalogue number. If plant specimens were collected from the field, describe the collection location, date and sampling procedures.*
Novel plant genotypes	*Describe the methods by which all novel plant genotypes were produced. This includes those generated by transgenic approaches, gene editing, chemical/radiation-based mutagenesis and hybridization. For transgenic lines, describe the transformation method, the number of independent lines analyzed and the generation upon which experiments were performed. For gene-edited lines, describe the editor used, the endogenous sequence targeted for editing, the targeting guide RNA sequence (if applicable) and how the editor was applied.*
Authentication	*Describe any authentication procedures for each seed stock used or novel genotype generated. Describe any experiments used to assess the effect of a mutation and, where applicable, how potential secondary effects (e.g. second site T-DNA insertions, mosiacism, off-target gene editing) were examined.*
